# Editorial: Liver Myofibroblasts

**DOI:** 10.3389/fphys.2016.00343

**Published:** 2016-08-05

**Authors:** Jiri Kanta, Alena Mrkvicová, Ralf Weiskirchen

**Affiliations:** ^1^Faculty of Medicine in Hradec Kralove, Charles UniversityPrague, Czech Republic; ^2^Institute of Molecular Pathobiochemistry, Experimental Gene Therapy and Clinical Chemistry, University Hospital RWTH AachenAachen, Germany

**Keywords:** myofibroblasts, liver fibrosis, hepatic stellate cells, portal fibroblasts, transdifferentiation, TGF-β, therapy

Myofibroblasts (MFB) were first identified in the granulation tissue of healing wounds. They have features of both fibroblasts and smooth muscle cells. In contrast to fibroblasts they contain cytoplasmic actin microfilaments (stress fibers) connected to the extracellular matrix (ECM) by focal contacts. MFB are connected to each other by adherens and gap junctions. Mechanical forces produced by their contraction facilitate wound closure. Prominent stress fibers can be used to identify MFB in the tissue. They are of mesenchymal origin and are produced by activation and transdifferentiation of quiescent cell precursors after tissue injury. They are not found in normal liver but they appear in large numbers in damaged liver and become a major source of ECM proteins that replace functional tissue. MFB precursors in the liver are hepatic stellate cells (HSC), portal fibroblasts, and circulating bone marrow-derived collagen-producing cells (fibrocytes). They may also arise in a process termed epithelial-to-mesenchymal transition in which epithelial cells acquire a mesenchymal phenotype. Based on the important role of MFB, the knowledge of the transdifferentiation process is critical to understanding the development liver fibrosis. Profibrogenic and proinflammatory cytokines produced by macrophages and T cells regulate fibrogenesis. TGF-β1, the main profibrogenic cytokine, is also produced by MFB and stimulates ECM production in an autocrine manner. Mechanical factors play a role in fibrosis development. Tissue tension facilitates TGF-β1 production and α-smooth actin expression, which in turn increases tension development. MFB are susceptible to apoptosis, their disappearance is important for fibrosis reversibility, and they may be a target of anti-fibrogenic therapy.

With the important role of MFB in the development of liver fibrosis in mind, leading experts in the field share their current perspectives on these cells in this Research Topic. As reviewed by Lepreux and Desmouliere, MFB are found in fetal liver and they reappear during liver injury. They are involved in tissue repair, in liver regeneration, and in liver cancer. HSC-derived MFB are studied in most cases. El Mourabit et al. have described a method to isolate MFB precursors from the rat biliary tree. These cells are highly proliferative and can be easily multiplied *in vitro*. Portal MFB differ in the expression of several genes from HSC-derived MFB, highlighting the distinct origin of the respective cell populations. Kawada concludes in his review that cytoglobin, a member of the mammalian globin family, is expressed in HSC and HSC-derived MFB but it is absent in MFB derived from portal fibroblasts. Therefore, cytoglobin may be used in future studies requiring the discrimination of both MFB subpopulations.

The review by Nwosu et al. shows that HSC transdifferentiation is accompanied by changes in the main metabolic pathways, glycolysis, tricarboxylic acid cycle, as well as in glutamine, fatty acid, and cholesterol metabolism. The authors demonstrate that the antioxidant defense system is also affected and that autophagy, the process of degradation of cellular organelles to generate energy, correlates with HSC activation. On the other hand, autophagy may protect from liver fibrosis in certain circumstances. Gene expression in HSC is also modulated by epigenetic mechanisms. Lambrecht et al. discuss a possible role of miRNAs in liver fibrosis. These short RNA molecules regulate gene expression both in normal and pathological conditions. Hypoxia in the liver cells that accompanies the development of fibrosis affects a number of miRNAs. Several miRNAs have been implicated in HSC activation.

Another review in the Research Topic highlights the role of NADPH oxidases in mediating activation of MFB and hepatic fibrogenesis. Chronic liver injury generates oxidative stress that leads to the damage of lipids, proteins, and DNA and necrosis and/or apoptosis of hepatocytes. Reactive oxygen species (ROS) stimulate the production of profibrogenic mediators by Kupffer cells and circulating inflammatory cells and activate HSC. NADPH oxidases are the source of ROS and thus play a role in HSC activation (Liang et al.).

Görtzen et al. have found that the activity of the GTPase RhoA increases in cirrhotic liver and in activated HSC which leads to decreased activity of the transmembrane protein c-SRC. As a consequence, HSC motility and migration is decreased in favor of contractility and ECM synthesis. The increasing tension of liver tissue in the proces of fibrosis can be mimicked by culturing cells on fibrinogen-coated polyacrylamide gels of variable stiffness.

Anti-fibrotic therapy may include protection of hepatocytes from apoptosis as the dying cells release signals recruiting immune cells to the sites of injury. According to the review by Wang et al., the blocking of TGF-β action, the elimination of activated HSC, and the inhibition of cholangiocyte proliferation are possible ways of anti-fibrotic treatment. Various chemicals of plant origin are tested as anti-fibrotic drugs. The hop constituent xanthohumol has been shown to inhibit HSC activation and hepatic carcinoma cell growth *in vitro*. However, as Weiskirchen and his associates discuss in their review, relatively large doses of xanthohumol need to be applied in animal experiments to inhibit pro-fibrogenic gene expression.

In conclusion, the articles in this Research Topic deal with the current topics of myofibroblast research—the origin of MFB in the fibrotic septa of cirrhotic liver, the regulation of the cell transdifferentiation and possible ways of fibrosis treatment. The editors hope that the Research Topic will help researchers to solve these problems.

## Author contributions

JK and RW contributed equally to the design of the manuscript, drafted the manuscript, and revised it. AM read the manuscript critically and approved it.

## Funding

Charles University grant PRVOUK P37/01 and DFG (SFB/TRR 57 P13 and Q3).

### Conflict of interest statement

The authors declare that the research was conducted in the absence of any commercial or financial relationships that could be construed as a potential conflict of interest.

